# The Significance of Observing the Risk of Non-communicable Diseases after Large-scale Disasters and Communicable Disease Epidemics

**DOI:** 10.31662/jmaj.2021-0126

**Published:** 2021-09-27

**Authors:** Hirohito Metoki

**Affiliations:** 1 Division of Public Health, Hygiene and Epidemiology, Tohoku Medical and Pharmaceutical University, Sendai, Japan; 2 Department of Preventive Medicine and Epidemiology, Tohoku Medical Megabank Organization, Tohoku University, Sendai, Japan

**Keywords:** Non-communicable diseases, COVID-19, disasters, cohort study, biobank

## Abstract

Non-communicable diseases (NCDs) are a global challenge, accounting for 71% of all deaths worldwide. The spread of coronavirus disease 2019 (COVID-19) and past huge disasters have affected the prevention and treatment of NCDs and require urgent action. In this narrative review, I will discuss several reports on the risk of NCDs during past disasters and propose possible future directions. Hypertension, the most common NCD, carries a high risk of death due to cerebrovascular, renal, and other complications. Effective use of information and communication technology-based telemedicine is necessary to manage the risk of cardiovascular diseases during disasters and pandemics. We observed the cumulative incidence of metabolic syndrome in subjects from tsunami-affected areas. We found that moving into prefabricated temporary housing was a risk factor for a higher incidence of metabolic syndrome in elderly females. Our follow-up of 1,009 subjects showed a slight, but significant, increase in HbA1c values after a state of emergency was introduced, even though the lockdown was not as stringent as in other countries. In a study elucidating the prevalence of wheezing and eczema symptoms and the associated factors after the Great East Japan Earthquake, psychological effects, such as depression and self-reported posttraumatic stress disorder, were observed, particularly in people with allergic diseases. In recent years, new birth cohort studies have been initiated to complement the studies designed to collect information across multiple generations, such as the Lifelines study in the Netherlands and the Avon Longitudinal Study of Parents and Children (ALSPAC) study in the UK. It is desirable to assess the effects of COVID-19 to complement the existing cohort studies in Japan as well.

## Introduction

Non-communicable diseases (NCDs) account for 71% of all deaths worldwide, presenting a challenge for the aging population and the world ^(1)^. According to a statement by World Health Organization, treatment and prevention of NCDs were affected by the spread of coronavirus disease 2019 (COVID-19), and urgent measures were required ^(2)^. Moreover, another large-scale disaster, the Great East Japan Earthquake (GEJE), occurred during the past decade. Many studies have reported that the risk of NCDs increases during large-scale disasters. In this narrative review, I explore some of the reports based on COVID-19 and GEJE parallelly and comment on the future directions.

## Disaster and Blood Pressure or Metabolic Syndrome

Hypertension, the most common NCD, is associated with a high risk of death due to cerebrovascular, cardiovascular, renal, and other complications ^(3)^. Therefore, preventing hypertension is essential. Sato et al. reported a rapid increase in blood pressure measured at home immediately after the GEJE. The difference in blood pressure and pulse rate measured at home between the day of the earthquake and the next day was 11.6/3.9 mmHg and 4.7 bpm, respectively ^(4)^. The standardized incidence rate (SIR) of cerebrovascular disease was 1.20 in the first four weeks after the earthquake but insignificant at other periods ^(5)^. SIR was higher in males (1.51), subjects aged over 75 years (1.35), and those residing in highly affected areas (1.35). The SIR of cerebral infarction in the first four weeks after the earthquake among males aged ≥ 75 years in the highly affected areas was 2.34. In other words, an over two-fold increase in the incidence of cerebral infarction among older males occurred in the first four weeks after the earthquake ^(5)^. Ishikuro et al. reported that pre-pregnancy body mass index (BMI) mediated the relationship between exposure to the GEJE and the prevalence of hypertensive disorders of pregnancy (HDP), and a decrease in the gestational week at delivery was observed with the presence of HDP ^(6)^. Narita et al. introduced an information and communication technology (ICT)-based blood pressure monitoring device in an evacuation center. They shared the patients’ blood pressure values through a database to support blood pressure management by remote monitoring and improve blood pressure control. They stated that the effective use of ICT-based telemedicine would be necessary for risk management of cardiovascular diseases during future disasters and pandemics ^(7)^.

The research project for the prospective investigation of health problems among survivors of the GEJE (RIAS Study) observed the cumulative incidence of metabolic syndrome in subjects from tsunami-affected areas. The study found that moving to prefabricated temporary housing was a risk factor for metabolic syndrome among elderly females ^(8)^.

Recently, an association between the spread of COVID-19 and metabolic syndrome and blood pressure has been reported. The effects of lack of exercise, overeating, irregular lifestyles, and stress were considered after social distancing was enforced. In a Brazilian cohort study, 43.1% of the 1,288 participants needed medical care, and 28.5% reported impaired NCD management during the social distancing period ^(9)^. Besides the changes in society and health care systems due to the COVID-19 pandemic, the incidence of diabetic ketoacidosis increased by 12%, and type 1 diabetes was more severe in newly diagnosed children ^(10)^. A follow-up of 435 subjects with relatively well-controlled type 1 and type 2 diabetes at Leiden University Medical Center showed increased perceived stress, anxiety, and weight, and decreased physical activity during the short-term lockdown measures, but glycemic control had not worsened ^(11)^. In contrast, our follow-up of 1,009 subjects showed that HbA1c values increased significantly from 7.45% to 7.53% after a state of emergency due to COVID-19 pandemic was declared. A deterioration in HbA1c values was observed among females, patients aged ≥ 65 years, those with a BMI of ≥ 25 kg/m^2^, and those not using insulin ^(12)^. Marcal et al. reported the need for exercise at home ^(13)^, but it was difficult to achieve due to the pandemic. Efforts to control the spread of COVID-19 have had a significant impact on the lifestyle of elderly participants in community-based exercise programs ^(14)^. Responses to a telephone survey among participants of the IMPACTS-BP (Implementation of Multifaceted Patient-Centered Treatment Strategies for Intensive Blood Pressure Control) study suggested that low-income patients, especially those who were black, were adversely affected by COVID-19 pandemic. Most patients who received necessary medical services were willing to return to their primary care clinic for hypertension management ^(15)^. Some diseases occurred more frequently, while others decreased following the stay-at-home order. A Japanese study based on the data from Fire and Disaster Management Agency database reported a decrease in the incidence of heatstroke due to a decreased exposure to risks following the stay-at-home order ^(16)^.

## Psychological Effects in Patients with Allergic Diseases

We investigated the prevalence of wheezing and eczema symptoms and associated factors after the GEJE. Younger age, history of hospitalization, and difficulties in children’s daily lives, as assessed by the Strengths and Difficulties Questionnaire, were significant predictors of allergic symptoms. Living in a coastal municipality was associated with eczema symptoms. Psychometric properties were also closely associated with allergic symptoms ^(17)^. Among boys, but not girls, experiencing the tsunami and living in a shelter were significant predictors of atopic dermatitis, while having their house destroyed and moving between houses were positively associated with a higher incidence of asthma among girls ^(18)^. A cross-sectional study that assessed the degree of depression symptoms and the risk of posttraumatic stress disorder (PTSD) in patients with allergic diseases during the COVID-19 pandemic found that psychological consequences, such as depression and self-reported PTSD, primarily affected the people with allergic reaction disorders ^(19)^.

## Impact of Disasters on Pregnant Women

Perinatal support seems essential when considering the impact of disasters. Intimate partner violence observed in affected areas may have lasted for several years after the GEJE ^(20)^. The incidence of postpartum depression in Miyagi Prefecture after the GEJE showed that exposure to the tsunami was significantly and independently associated with an Edinburgh Postnatal Depression Scale score of ≥ 9. We previously suggested that postpartum women and their children should be treated as a vulnerable group, and protective systems should be established to prepare them for similar disasters in the future ^(21)^.

The COVID-19 pandemic might have significantly impacted maternal mental health. Feelings of anxiety and depression were associated with maternal anxiety about possible vertical transmission of the virus to their infants, limited access to perinatal care resources, and lack of social support ^(22), (23)^. A cross-sectional study conducted during the stay-at-home order found that unhealthy lifestyles involving smoking and drinking habits and being a female were important risk factors for depressive symptoms, whereas having exercise habits and someone to consult about worries were protective factors ^(24)^. A short report suggested that staying connected was important even when staying at home ^(25)^. Pregnant women with COVID-19 reportedly had a disproportionately poor socioeconomic status, regardless of the economic status of the country ^(26)^. These reports were also a source of stress for pregnant and postpartum women without COVID-19 ^(27), (28)^. Pregnant women and new mothers were more likely to experience mental illness than non-pregnant women ^(29)^. Social distancing, isolation, and quarantine procedures implemented during the pandemic increased the risk of psychological problems in pregnant women and new mothers ^(22), (23), (28)^. A longitudinal study of emergency department visits in the United States reported increases in mental health conditions, suspected child abuse and neglect, drug overdose, and suicide attempts, but not intimate partner violence ^(30)^. In Japan, the Domestic Violence Consultation Plus helpline system was in contact with shelters throughout the country to help enact safety measures against COVID-19 ^(31)^.

An online survey was conducted during the COVID-19 pandemic from May 19 to June 6, 2020, in which 292 pregnant Japanese women and 13 Japanese women undergoing fertility treatments completed the Japanese version of the Fear of COVID-19 Scale and reported the risk factors for mental illness ^(32)^. The pregnant Japanese women scored higher on the scale than infertile patients. The Fear of COVID-19 Scale score among pregnant women who focused on websites and social networking sites was positively associated with stockpiling and health monitoring and negatively associated with the fear of COVID-19. Pregnant women are highly anxious, and websites and social networking sites may have effectively decreased their anxiety. The authors suggested that it is important to disseminate accurate information on preventing infectious diseases and how to ease anxiety in pregnant women ^(32)^. Being physically away from the office due to teleworking may not reduce pregnancy discrimination and depression. To protect pregnant women’s mental health and employment, employers should comply with the law and take measures to prevent pregnancy discrimination ^(33)^. Non-adherence to all social distancing rules had a strong association with vulnerability to COVID-19. People living in high-risk environments such as multiple-occupancy houses should be supported, especially when asked to stay at home. A public health messaging service should emphasize shared responsibility and public consciousness ^(34)^.

## Approach to Observation Based on Existing Large Cohort Studies

Recently, new birth cohort studies have been initiated to complement the Lifelines Cohort Study in the Netherlands and the Avon Longitudinal Study of Parents and Children (ALSPAC) study in the UK, which have already collected information over multiple generations. Studies investigating the spread of COVID-19, its social impact, and its impact on NCD risk are being conducted to complement these cohort studies.

In the ALSPAC study, depression levels during the pandemic were similar to pre-pandemic levels in the participants born in 1990, but the number of people feeling anxious almost doubled to 24%, compared to 13% before the pandemic. Moreover, anxiety and depression during the pandemic were prevalent in both generations (participants born in 1990 and their parents) among younger people, females, those with preexisting mental or physical conditions, and those disadvantaged socioeconomically, even after controlling for pre-pandemic anxiety and depression ^(35)^.

The Lifelines Cohort Study sent out questionnaires biweekly starting in March 2020 and monthly from July 2020, with new rounds of questionnaires continuing through early 2021 ^(36)^. The cohort data were used to address how the COVID-19 pandemic developed in the northern provinces of the Netherlands, what environmental and genetic risk factors predicted disease incidence and severity, and the psychological and social impacts of the crisis ^(36)^. After adjusting for age, sex, lifestyle, BMI, and ethnicity, participants with lower education or a lower income were less likely to self-report the infection and be tested than the more educated and higher-income groups ^(37)^. In a study on data from the UK Biobank, high social deprivation was a continuous risk factor for death from COVID-19, with the most disadvantaged 20% having a two- to threefold higher risk than the most advantaged 20% ^(38)^. Given the similarity of social gradients shared by COVID-19, influenza, and cardiovascular disease, better social policies are essential for reducing the health care burden ^(38)^.

## Perspectives

New cohort studies after a disaster could help identify new indicators, while ongoing cohort studies initiated before a disaster occurred could help minimize bias regarding survivors’ pre-disaster information ^(39)^. As Tsuboya et al. summarized, research, constantly improved by new data, is needed to characterize vulnerable populations, save lives, and reduce the damage that can be caused by disasters in the future ^(39)^. Epidemiological and clinical studies require long-term investigations of large populations. In Europe and the United States, significant time, personnel, and research funds have been invested in these studies, but the resources had not been sufficient in Japan. Moreover, during the COVID-19 pandemic, additional surveys were actively planned and conducted in established cohorts. It is desirable to assess the effects of COVID-19 to complement existing cohort studies in Japan. Recently, a large-scale genomic cohort of 366,000 people was constructed in Japan ^(40)^. Including information for morbidity and preventive behaviors while addressing ethical, legal, and social issues, and to use it as a basis for designing countermeasures against communicable diseases and NCDs in the future, are recommended.

## Article Information

This article is based on the study, which received the Medical Research Encouragement Prize of The Japan Medical Association in 2020.

### Conflicts of Interest

HM concurrently hold the non-compensated sub-directorship at the Tohoku Institute for Management of Blood Pressure, which is supported by Omron Health care Co. Ltd, and involved in collaborative research with Omron Health care in another study. H.M. has also received grants or scholarship from Academic Contributions from Pfizer Japan Inc., Astellas Research Support, Daiichi Sankyo Co., Ltd. Bayer Academic Support, Otsuka Pharmaceutical Co., Ltd, Takeda Research Support, Eli Lilly Japan K.K., Baxter Co., Ltd., Mitsubishi Tanabe Pharma Corporation, Chugai Pharmaceutical Co., Ltd., Teijin Pharma Limited. These companies were not involved in this review article.

### Sources of Funding

This work was supported by Medical Research Encouragement Prize of The Japan Medical Association

### Approval by Institutional Review Board (IRB)

Since this narrative review is based on previously published articles, IRB approval is not required.
